# Knee alterations in rheumatoid arthritis: Comparison of US and MRI

**DOI:** 10.1515/med-2021-0310

**Published:** 2021-06-24

**Authors:** Lu Xiao, Yanyan Huang, Feng Zhan

**Affiliations:** Department of Rheumatology, Hainan General Hospital (Hainan Affiliated Hospital of Hainan Medical University), Hainan 570311, China

**Keywords:** ultrasonography, rheumatoid arthritis, MRI, anti-cyclic citrullinated peptide antibody

## Abstract

**Objective:**

This study was designed to compare the diagnostic efficacy of ultrasonography (US) and magnetic resonance imaging (MRI) in detecting changes in the knee of patients with rheumatoid arthritis (RA) and discover the possible association between the serological index and bone erosion detected by US.

**Patients and methods:**

In this retrospective study, the US images and MRI findings of the knee in patients with RA from December 2017 to January 2020 were evaluated. Diagnostic outcomes were compared. The rheumatoid factor, C-reactive protein, erythrocyte sedimentation rate, and anti-cyclic citrullinated peptide antibody (ACPA) levels of the patients were recorded. The relation between laboratory index and US findings was analyzed by multivariable logistic regression.

**Results:**

US showed remarkable accuracy, sensitivity, and specificity in diagnosing synovitis, bone erosion, and soft tissue swelling. In terms of reliability, the agreement between US and MRI was moderate to almost perfect. Meanwhile, a positive association between ACPA level and bone erosion was observed in patients with RA.

**Conclusions:**

US may have a role as the initial imaging modality in patients with RA. Patients with higher ACPA levels may need more active treatment because they are more likely to have bone erosion detected by US.

## 1 Introduction

Rheumatoid arthritis (RA), a chronic systemic autoimmune disease of unknown etiology, affects approximately 1% of the world population, 50% of whom being unable to work within 10 years of the disease onset [1]. Meanwhile, RA is the most prevalent inflammatory arthropathy that is responsible for joint structural destruction [2]. Therefore, early diagnosis to optimize the tight control of disease activity seemed to be notably important to reduce joint structural damage [3,4].

In daily clinical practice, conventional radiography remains the mainstay for the evaluation of patients with RA [5]. However, X-ray examination could only show late bone deformity [6]. Nowadays, magnetic resonance imaging (MRI) and ultrasonography (US) are gradually applied in clinical practice to detect early changes and inflammation in the joints of patients with RA. US is actively applied in disease management and early diagnosis, as it is safe, noninvasive, and low-cost, and contains non-ionizing radiation. Several studies have shown that US can detect more erosion than X-ray, especially at the early stage of RA [7]. High-resolution musculoskeletal US can demonstrate consistent and reproducible results according to trained rheumatologists [8]. Despite increasing efforts on the validation and reliability of US in the evaluation of small joints, evidence for larger joints, such as the knee, is still limited [9]. Although RA mostly influences the small joints in the hands and wrist, it also affects larger joints as the disease progresses; the knee is influenced in 90% of patients [10]. Knees are vital weight-bearing joints, and the limitation of motion in the knee joint will cause severe disability. Hence, discovering early changes in the knee joint during the disease course via a more accessible way is of great importance. In this study, we aimed to determine the accuracy and consistency of US in discovering knee changes in patients with RA and compare the diagnostic performance of US with that of MRI to offer a more convenient method in the early diagnosis field.

The frequently used serological indicators of RA include erythrocyte sedimentation rate (ESR), C-reactive protein (CRP), rheumatoid factor (RF), and anti-citrullinated peptide antibodies (ACPA). Among them, ACPA is proven to be the main predictive factor of bone erosion on plain film [11]. However, the link between serological parameters and the bone erosion detected by US has not been discussed previously. Bone erosion may consequently result in joint deformity. Therefore, figuring out the possible factors associated with bone erosion is very meaningful in the identification of high-risk individuals and may have implications for patient information and management.

In conclusion, our research plans to determine the accuracy of US in evaluating knee joint pathologies and the possible association between serological parameters and the bone erosion detected by US to provide possible predictors of disease progression.

## 2 Patients and methods

### 2.1 Patient selection

In this retrospective study, one hundred patients diagnosed with RA according to the 2010 American College of Rheumatology/European League Against Rheumatism Classification and Scoring Criteria in the Outpatient and Inpatient Department of Rheumatology of Hainan General Hospital from December 2017 to January 2020 were enrolled [12]. Exclusion criteria included the following: younger than 18 years of age, severe knee joint deformity, and history of knee surgery/trauma and bacterial infection (such as purulent arthritis) in the knee.


**Ethics approval and consent to participate:** This research was approved by the ethics committee of Hainan General Hospital. Subjects were informed of and provided written consent to the experimental protocol and procedures. The ethical approval number was MED-ETH-RE [2020]12.
**Consent for publication:** Informed consent was obtained from all patients before they participated in the study.

### 2.2 Clinical data and laboratory measures

We collected demographic data of each patient, including age and mean disease duration. Blood samples were obtained from an antecubital vein in the morning to determine CRP, ESR, RF, and ACPA levels.

### 2.3 US and MRI data

Philips high-grade color ultrasonic diagnostic apparatus and a 10–18 MHz linear array probe were used for ultrasonic investigation. An US doctor who had more than 5 years of experience and was blinded to the clinical examination and MRI findings conducted the investigation. A fixed protocol following the technical guidelines of the European Society of Musculoskeletal Radiology for knee scanning was applied [13]. Patients were laid in a supine position with knees flexed to 60° to explore the different parts of the knee. The US classification standards of different pathologies were followed according to the Outcome Measures in Rheumatoid Arthritis Clinical Trials US group definition [14]. Inflammatory and structural changes were graded as present or absent (score 0 or 1). The results of US were read by one radiologist and one rheumatologist who were blinded to the clinical diagnosis and MRI findings. When their conclusions were different, the final US diagnosis was made by consulting the director of US.

MRI scans were made using a 3.0T MRI scanner (GE Signa Horizon Echospeed, LX9.0, General Electric Medical Systems, Milwaukee). The MRI sequences were chosen in close collaboration with an MRI technician and musculoskeletal radiologist. Coronal, sagittal, and axial T1-weighted fat-suppressed pre-/postintravenous gadolinium (0.1 mmol Gd/kg body weight; Magnevist, Bayer Schering Pharma AG, Leverkusen, Germany) images were acquired through 3D dual-echo technique. Patients were in a supine position with the knee joint extension placed in a dedicated extremity coil centrally in the magnetic field. Inflammatory and structural changes were graded as present or absent (score 0 or 1). The MRI results were read by one radiologist and one rheumatologist who were blinded to the clinical diagnosis and US findings. When their conclusions were different, the final MRI diagnosis was made by consulting the director of radiology.

The presence of synovitis, ligament injury, tenosynovitis, joint space narrowing, bone erosion, and soft tissue (including the periarticular muscle, ligament, and tendon) swelling was analyzed by US and MRI.

### 2.4 Statistical analysis

SPSS 20.0, statistical software, was used to analyze the data. Quantitative clinical and demographic variables are reported as mean ± standard deviation. The comparison of the quantitative values was performed with Student’s *t*-test for paired samples, and the McNemar test was used for qualitative values. The results of US and MRI diagnosis were considered dichotomous data (absent/present). Reliability is quantified by the value of kappa (<0 means poor agreement, 0.0–0.2 means slight agreement, 0.21–0.40 means fair agreement, 0.41–0.60 means moderate agreement, 0.61–0.8 means substantial agreement, and 0.81–1.00 means almost perfect agreement). The association between the laboratory parameters and US findings was analyzed by multivariable logistic regression. *P* < 0.05 was considered to be significant.

## 3 Results

### 3.1 Demographic characteristics of patients

One hundred patients (10 males and 90 females) with RA who consulted Hainan General Hospital from December 2017 to January 2020 and met our inclusion criteria were included in this study. The demographic data of the patients are summarized in Table 1. The mean age was 53.5 ± 9.5 years. The mean disease duration was 15.5 ± 7.3 months. The mean levels of CRP, ESR, RF, and ACPA were 45.5 ± 38.1 mg/L, 66.5 ± 35.3 mm/h, 254.7 ± 237.1 IU/mL, and 99.8 ± 86.2 IU/mL, respectively.

**Table 1:** Demographics and clinical characteristics of the patients

Patient characteristics	Results
Gender (male/female)	10/90
Mean age (years)	53.5 ± 9.5
Mean duration of disease (months)	15.5 ± 7.3
CRP (mg/L)	45.5 ± 38.1
ESR (mm/h)	66.5 ± 35.3
RF (IU/mL)	254.7 ± 237.1
CCP (IU/mL)	99.8 ± 86.2

Data presented as mean ± SD or number of patients, CRP: C-reactive protein, ESR: erythrocyte sedimentation rate, RF: rheumatoid factor, CPC: anti-cyclic citrullinated peptide antibody.

### 3.2 Comparison of US and MRI of knee joint examination in the diagnosis of patients with RA

**Figure 1:**
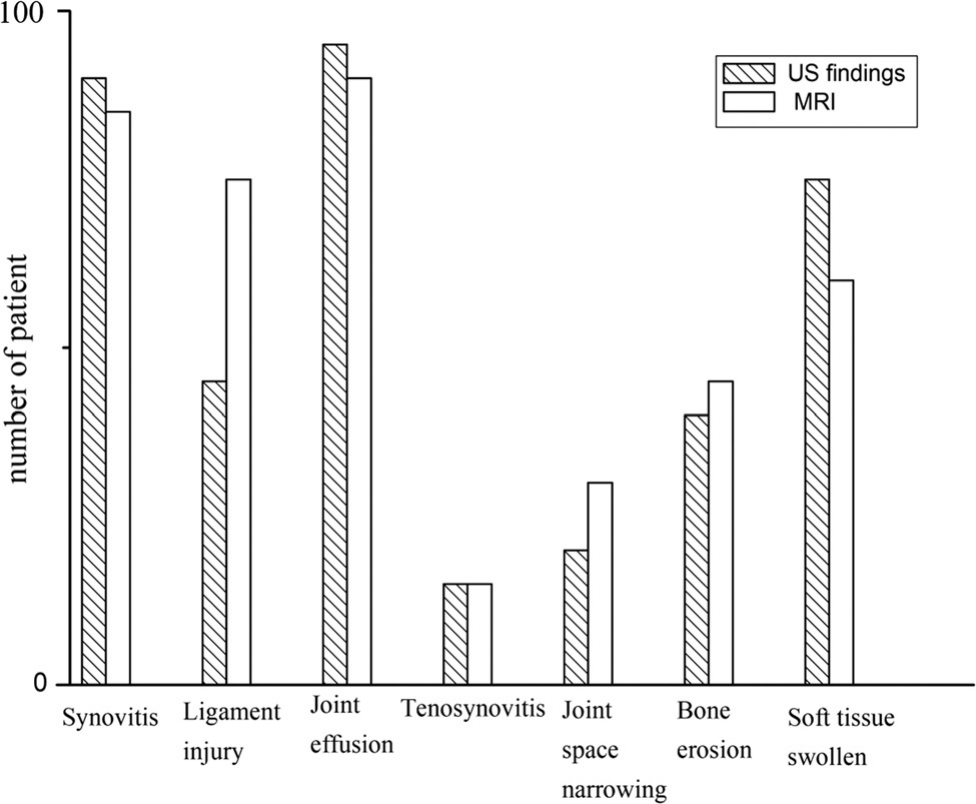
US and MRI findings of patients with RA.

As shown in Figure 1, the common abnormalities detected by US were joint effusion (95%), synovitis (90%), soft tissue swelling (75%), ligament injury (45%), bone erosion (40%), joint space narrowing (20%), and tenosynovitis (15%). The common pathologies detected by MRI were joint effusion (90%) synovitis (85%), ligament injury (75%), soft tissue swelling (60%), bone erosion (45%), joint space narrowing (30%), and tenosynovitis (15%). The diagnostic performance of US and MRI in evaluating the pathological changes in knee joints was compared. As shown in Table 2, US had high sensitivity and specificity in bone erosion, joint space narrowing, and synovitis detection.

**Table 2:** Comparison of ultrasound versus MRI in detecting knee pathologies

US findings	Sensitivity (%)	Specificity (%)	PPV (%)	NPV (%)
Synovitis	94.4	66.7	66.7	50
Ligament injury	66.7	100	100	44.4
Joint effusion	100	50	95.4	50
Tenosynovitis	66.7	94.4	66.7	94.4
Joint space narrowing	66.7	100	100	78.7
Bone erosion	88.9	100	100	91.6
Soft tissue swelling	100	62.5	80	100

PPV, positive predictive value; NPV, negative predictive value.

### 3.3 Comparison of US and MRI in the evaluation of the different knee pathologies in patients with RA

The reliability of US compared with MRI findings was measured using kappa agreement, and the results are shown in Table 3. US revealed at least moderate agreement with MRI in diagnosing different lesions of the knee joints in the patients with RA. The agreement in the diagnosis of synovitis (substantial, kappa = 0.78, *P* < 0.05), ligament injury (moderate, kappa = 0.43, *P* < 0.05), joint effusion (substantial, kappa = 0.64, *P* < 0.05), tenosynovitis (substantial, kappa = 0.61, *P* < 0.05), joint space narrowing (substantial, kappa = 0.74, *P* < 0.05), bone erosion (almost perfect, kappa = 0.89, *P* < 0.05), and soft tissue swelling (substantial, kappa = 0.67, *P* < 0.05) is listed in Table 3.

**Table 3:** Comparison of ultrasound and MRI in the evaluation of different knee pathologies

Knee pathology	US (*n*)	MRI (*n*)	kappa	*P* value
Synovitis	90	85	0.78	<0.001
Ligament injury	45	75	0.43	0.020
Joint effusion	95	90	0.64	0.002
Tenosynovitis	15	15	0.61	0.007
Joint space narrowing	20	30	0.74	0.001
Bone erosion	40	45	0.89	<0.001
Soft tissue swelling	75	60	0.67	0.002

### 3.4 Correlation between US detecting bone erosion and different parameters

Multivariable logistic regression was applied to clarify the relation between the level of laboratory parameters and US-detected bone erosion. The results are shown in Table 4. Statistically significant associations were found between ACPA levels and the bone erosion detected by US (odds ratio = 1.017, 95% confidence interval = 1.001–1.033; *P* = 0.038).

**Table 4:** Correlation between the US detecting bone erosion and different parameters

	*P*-value	OR	95% CI
Duration of disease	0.473	1.002	0.996–1.008
CCP	0.038*	1.017	1.001–1.033
RF	0.402	1.005	0.993–1.018
ESR	0.210	0.962	0.905–1.022
CRP	0.412	1.031	0.959–1.107

CPC: anti-cyclic citrullinated peptide antibody, RF: rheumatoid factor, ESR: erythrocyte sedimentation rate, CRP: C-reactive protein, OR: odds ratios, CI: confidence intervals, *P < 0.05.

## 4 Discussion

RA is a chronic autoimmune disease characterized by synovitis and bone erosion. RA always has an agonizing long-term course in patients who are not diagnosed and treated in time. Thus, early diagnosis to optimize the tight control of disease activity is notably important. This study aimed to determine the diagnostic efficacy of US compared with MRI in detecting knee changes in patients with RA, and the possible association between serological parameters and the bone erosion detected by US to provide a possible predictor of RA progression. Several important results of this research, which might have implications in clinical practice and future research, should be paid attention to. First, our study determined that US may play an important role in the initial imaging modality of RA. US has satisfactory accuracy compared with MRI in detecting different knee lesions. Second, patients with higher ACPA levels are more likely to have bone erosion detected by US. Therefore, these patients may need to be monitored more intensively. The results may have implications for RA information and management.

US displays a remarkable performance in diagnosing inflammatory lesions and assessing structural damage with the development of high-frequency US technology [15,16]. US offers many advantages, such as accessibility, low cost, and lack of irradiation [17]. It demonstrates good intra- and inter-reliability in the diagnosis of RA and other musculoskeletal diseases [17,18,19]. In the present research, we found that US showed satisfactory accuracy in diagnosing synovitis, bone erosion, and soft tissue swelling when compared with MRI. Kappa agreement was conducted to evaluate the consistency of the results between US and MRI. The agreement between US and MRI was substantial to almost perfect in detecting synovitis, ligament injury, tenosynovitis, joint space narrowing, bone erosion, and soft tissue swelling. Previous studies that also focused on the accuracy of US drew the same conclusions as ours. A study conducted in 2019 reported that US is an accurate and reliable tool in detecting shoulder pathologies [20]. Luminița and his colleagues provided the same conclusion about the ankle [21]. Thus, US may play an important role in the initial imaging modality of the knee in patients with RA.

In the meantime, we tried to discover the possible association between serological parameters and the bone erosion detected by US. We found a positive association between ACPA level and bone erosion. The presence of ACPA is the main predictive factor of bone erosion in radiographs [12]. However, the possible association with US has not been demonstrated. ACPA, as a polypeptide fragment of cyclic filaggrin, is an IgG type that is considered an ideal serological marker for RA diagnosis because of its high sensitivity and specificity [22,23]. In 2019, some experts proved that the presence of ACPA is associated with new bone erosion in patients with RA treated with disease-modifying antirheumatic drugs. In 2018, an 11-year cohort study has shown that the baseline level of ACPA predicts erosive progression. These outcomes partly agree with ours. Furthermore, ACPA is regarded as an essential marker related to bone loss and alterations in skeletal metabolism even before the clinical onset of RA [24]. Haag et al. demonstrated that ACPA contributes to the induction of joint inflammation in RA [25]. Consequently, ACPA may play a role in joint bone erosion. Hence, patients with higher ACPA levels could suffer from bone erosion earlier which indicated that these patients need to be monitored more intensively.

In the interpretation of our results, the following limitations require careful discussion. First, only one hundred patients with RA were recruited. The modest sample size might increase the possibility of type II error. A larger group of patients would probably strengthen the results. Second, our study is a retrospective study; therefore, the patients’ Disease Activity Score-28 for RA was difficult to obtain for clinical comparison. As a result, a more precisely designed prospective study is needed to further assess these potentially promising findings.
